# Characterization and reverse genetic establishment of cattle derived Akabane virus in China

**DOI:** 10.1186/s12917-021-03054-x

**Published:** 2021-11-15

**Authors:** Dongjie Chen, Di Wang, Fang Wei, Yufang Kong, Junhua Deng, Xiangmei Lin, Shaoqiang Wu

**Affiliations:** 1 Institute of Animal Inspection and Quarantine, Chinese Academy of Inspection and Quarantine, Beijing, 100176 China; 2 School of Agroforestry and Medicine, Open University of China, Beijing, 100039 China

**Keywords:** Akabane virus, Isolate, Phylogenetic analysis, Reverse genetic system

## Abstract

**Background:**

Akabane virus (AKAV) is an important insect-borne virus which is widely distributed throughout the world except the Europe and is considered as a great threat to herbivore health.

**Results:**

An AKAV strain defined as TJ2016 was firstly isolated from the bovine sera in China in 2016. Sequence analysis of the S and M segments suggested that the isolated AKAV strain was closely related to the AKAV strains JaGAr39 and JaLAB39, which belonged to AKAV genogroup II. To further study the pathogenic mechanism of AKAV, the full-length cDNA clone of TJ2016 S, M, and L segment was constructed separately into the TVT7R plasmid at the downsteam of T7 promoter and named as TVT7R-S, TVT7R-M, and TVT7R-L, respectively. The above three plasmids were further transfected into the BSR-T7/5 cells simultaneously with a ratio of 1:1:1 to produce the rescued virus AKAV. Compared with the parental wild type AKAV (wtAKAV), the rescued virus (rAKAV) was proved to be with similar cytopathic effects (CPE), plaque sizes and growth kinetics in BHK-21 cells.

**Conclusion:**

We successfully isolated a AKAV strain TJ2016 from the sera of cattle and established a reverse genetic platform for AKAV genome manipulation. The established reverse genetic system is also a powerful tool for further research on AKAV pathogenesis and even vaccine studies.

**Supplementary Information:**

The online version contains supplementary material available at 10.1186/s12917-021-03054-x.

## Background

Akabane disease (AKA) is an important arthropod-borne disease of cattle and sheep, which is characterized by abortion, premature birth, stillbirth and congenital arthrogryposis hydranencephaly (AH) syndrome [1, 2]. To date, AKA is widely distributed throughout the world except the Europe and has caused serious economic losses to animal husbandry, which poses a great threat to the cattle and sheep breeding industry [3–5].

As the pathogen of AKA, Akabane virus (AKAV) is an orthobunyavirus which is biological transmitted between susceptible vertebrate hosts primarily by hematophagous arthropods [1]. Since AKAV was firstly isolated in Japan from the mosquitoes *Aedes vexans* and *Culex tritaeniorhynchus* in 1959, it has been widely detected in Australia, Asia, and Africa [6]. AKAV is an enveloped virus with three segments (S, M, and L) of single-stranded negative-sense RNA genome and encodes four structural proteins: two virion glycoproteins (Gn and Gc) on the M segment, and two internal virion components, the nucleocapsid (N) protein on the S segment and the viral RNA-dependent RNA-polymerase (L protein) on the L segment [7]. In addition, two non-structural proteins NSm and NSs are encoded by the M and S segment, respectively. According to the phylogenetic analysis of S segment, AKAVs are divided into four genetically distinct groups (genogroups I-IV) and genogroup I is further subdivided into two subgroups (Ia and Ib) [8]. In China, a serological survey of AKAVs in Xinjiang province indicated that the neutralizing antibody positive rate of AKAV in cattle and sheep was 20.32 and 18.15%, respectively [9]. A large-scale serological survey of AKAV from 2006 to 2015 in 24 provinces of China indicated that the overall seroprevalence rate for AKAV antibodies was 21.3% in cattle and 12.0% in sheep or goats [10]. Furthermore, some AKAV isolates have also been reported in China. In July to August of 2010 and in August of 2011, six AKAVs were isolated from *culex quinquefasciatus* and *anopheles sinensis* in Hunan province [11]. In 2013-2016, five novel AKAVs were isolated from bamboo rats in Guangxi, China [12]. Moreover, a AKAV was isolated from a sentinel goat in Guangxi province of China in 2016 [13]. However, there has never been a report of direct isolation of AKAV from cattle in China so far. In the present study, we described the first cattle-derived AKAV isolate TJ2016 in China and investigated the genetic diversities of the current isolate with the previously reported AKAV isolates. Then, we established the reverse genetic system based on cattle-derived AKAV isolate, which was a useful tool for studying the basic mechanism of AKAV pathogenesis.

## Results

### Phylogenetic analysis of the S and M segments

**Fig. 1:**
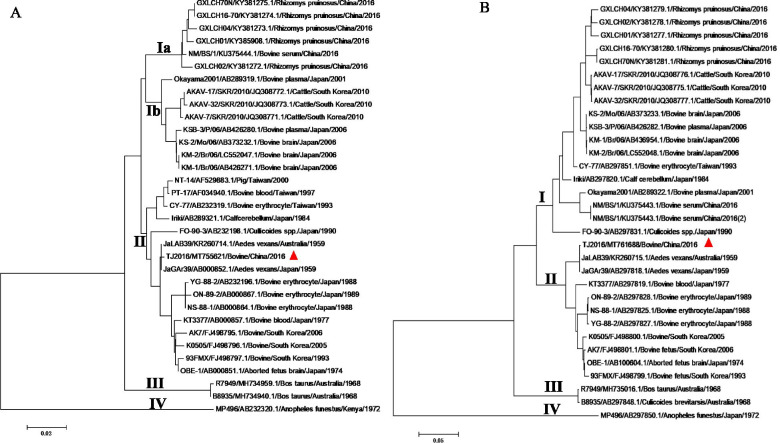
Phylogenetic tree of AKAV based on the CDS of the S (**A**) and M (**B**) segments. Sequence of the AKAV TJ2016 was indicated by red triangles (  ). The nucleotide sequences of other AKAV strains used in this study were obtained from GenBank and given in Table 2

For the phylogenetic analysis of the isolated AKAV TJ2016, nine pairs of primers were designed based on the published genome sequence of AKAV strain DHL10M110 (KY284023.1, KY284022.1, KY284021.1) and were used to amplify the whole genome of AKAV isolate (Table 1, Number 1 to 18). After sequencing, the S, M, and L segment of TJ2016 was 856, 4309, and 6868 bp in length, respectively. The sequences of S, M, and L segment were submitted to the GenBank (GenBank No. MT755621, MT761688 and MT761689). Then, the phylogenetic trees were constructed to clarify the genetic relationships between TJ2016 and the AKAV strains reported previously. In this assay, the intact open reading frames (ORFs) of S and M segments alignment were performed by Clustal W method using MEGA5.0 software, and the phylogenetic trees were generated for each segment by the Neighbor-Joining (NJ) method. As shown in Fig. 1A, the phylogenetic itree based on the sequences of S ORF revealed that AKAV isolates were segregated into four distinct genogroups (I, II, III and IV). Among them, genogroup I was further divided into subgroup Ia and Ib. The isolated AKAV TJ2016 was most closely related with two Japanese and Australian strains isolated in 1959, and all belonged to genogroup II. The identities of the TJ2016 isolate to the JaGAr39 and JaLAB39 were both 99.95% at nucleotide level. On the other hand, the phylogenetic tree based on M ORF revealed that AKAV TJ2016 also belonged to genogroup II together with the Japanese and Australian JaGAr39 and JaLAB39 strains (Fig. 1B), and the identities were both 99.96% at nucleotide level.

**Table 1:** Primers used in this study

Number	Name	Sequences	Located sites (bp)
1	AKAV-S-F	AGTAGTGAACTCCACTATTAACTACGC	1-27
2	AKAV-S-R	AGTAGTGTGCTCCACTAATTAACTATAAAC	827-856
3	AKAV-M1-F	AGTAGTGAACTACCACAACAAAATGATT	1-28
4	AKAV-M1-R	CTTGTATGCAAGCACTAAAAGC	1409-1432
5	AKAV-M2-F	CTAGATAATTTCACATCTCATTGCC	1253-1277
6	AKAV-M2-R	TTTACTCTGGAAATAACTGTTGCTTC	3182-3207
7	AKAV-M3-F	CAAGATTCAAGACAGCTACATAAC	2830-2853
8	AKAV-M3-R	AGTAGTGTTCTACCACAACAAATAATTATT	4280-4309
9	AKAV-L1-F	AGTAGTGTACCCCTAAATACAACATAC	1-27
10	AKAV-L1-R	CATATTTGGCTTTGATAATATCTTGTCAAC	1399-1428
11	AKAV-L2-F	CGAAGCTATAAAAATTGGTACCTC	1167-1190
12	AKAV-L2-R	CCATCTCCAGGTTCGCTAATCATCTCATCTG	2975-3005
13	AKAV-L3-F	CGAGCATATACTCCAAGTCATGAAAAATC	2808-2836
14	AKAV-L3-R	TCATTCCGTTACGATCCATTTG	4777-4798
15	AKAV-L4-F	GAAGACCTTTGTTGAGACGTATCGACAG	4520-4647
16	AKAV-L4-R	GCATCAATTTTGAAAGATCTATACCCCC	6040-6097
17	AKAV-L5-F	GGATAGAATAGAAATGCTCAATATCG	5937-5962-
18	AKAV-L5-R	AGTAGTGTGCCCCTAAATGCAATAATATAC	6839-6868
19	rAKAV-S-F	GGGGTACC*CGTCTC*ATATAGAGTAGTGAACTCCACTATTAACTACGC	1-27
20	rAKAV-S-R	GCTCTAGA*CGTCTC*TACCCAGTAGTGTGCTCCACTAATTAAC	834-856
21	rAKAV-M-F	GGGGTACC*CGTCTC*ATATAGAGTAGTGAACTACCACAACAAAATG	1-25
22	rAKAV-M-R	GCTCTAGA*CGTCTC*TACCCAGTAGTGTTCTACCACAACAAATAATT	4283-4309
23	rAKAV-L-F	GGGGTACC*CGTCTC*ATATAGAGTAGTGTACCCCTAAATACAACATAC	1-27
24	rAKAV-L-R	GCTCTAGA*CGTCTC*TACCCAGTAGTGTGCCCCTAAATGCAATAATATAC	6838-6868

### Reverse genetic rescue of AKAV TJ2016

**Fig. 2:**
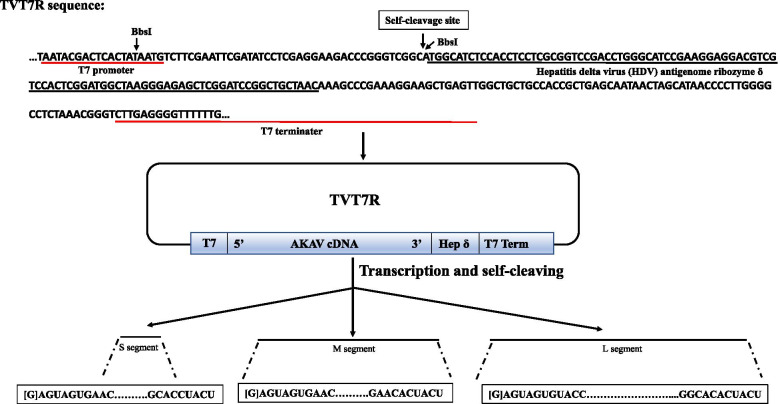
Simplified scheme for the construction of TVT7R series. The upper part of the figures shows the sequence around *Bbs*I restriction sites of TVT7R that is used to insert AKAV S, M or L segment. RNA transcripts produced by bacteriophage T7 RNA polymerase would contain one G residues, derived from the cloning site, before the authentic AKAV 5′ terminal sequence. The exact 3′ end of the transcript RNA was specified self-cleavage by the hepatitis delta virus (HDV) antigenome ribozyme. The conserved terminal bases of all three AKAV segments are shown. T7, T7 promoter; T7 term, T7 transcription termination sequence

**Fig. 3:**
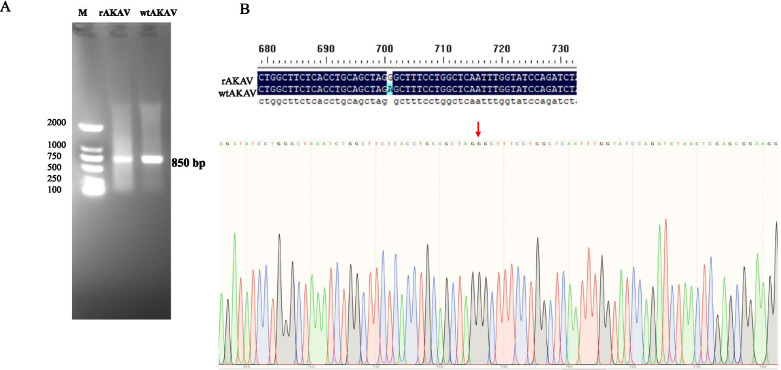
PCR amplification and sequencing identification of rAKAV

The simplified scheme for the construction of plasmids containing full-length of AKAV cDNA was shown in Fig. 2. The plasmid TVT7R which serves as a basis for reconstruction of AKAV cDNA could be cleaved between the T7 promoter and the Hepatitis delta virus (HDV) antigenome ribozyme with the endonuclease *Bbs*I, generating noncompatible sticky ends, which were used to insert the S, M, and L segments. The full-lengths of AKAV TJ2016 S, M, and L segments were amplified by KOD Fx Neo polymerase (ToYoBo, Osaka, Japan) using the gene-specific primers in Table 1 (Number 19 to 24) and digested using endonuclease *BsmB*I. Then, the generated compatible sticky ends with TVT7R plasmid were integrated through T4 ligase. After transcription and self-cleaving in the BSR-T7/5 cells, the exact 3′ end of the RNA was specially cleaved by HDV antigenome ribozyme and the genome of AKAV TJ2016 is generated with an excess G nucleotide at the 5′ end. To distinguish the rescued AKAV (rAKAV) with wild type AKAV (wtAKAV), a synonymous mutation (A702G) was generated in S segment and retained as a genetic marker. We transfected the plasmids TVT7R-AKAV-S, TVT7R-AKAV-M and TVT7R-AKAV-L (1:1:1) into BSR-T7/5 cells at a confluency of 80-90% in six-well plates. After three times of plaque purification, AKAV RNA was extracted and amplified using the primers AKAV-S-F and AKAV-S-R in Table 1. As shown in Fig. 3A, the fragment of approximately 850 bp was amplified successfully in both rAKAV and wtAKAV, and the nucleic acid sequencing proved that a synonymous mutation (A702G) was found in the S segment of rAKAV distinguished from wtAKAV (Fig. 3B). In addition, the full-length sequencing of rAKAV proved that no other nucleic acid mutation besides the synonymous mutation existed in the S segment.

### Cytopathic effect (CPE) and indirect immunofluorescence identification

**Fig. 4:**
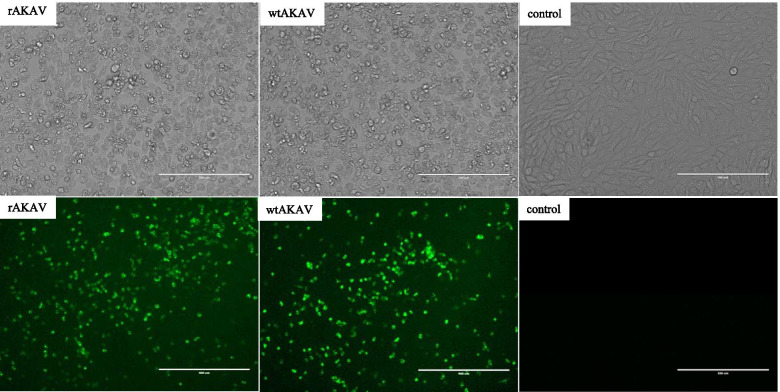
CPE and IFA identification of rAKAV. Indirect immunofluorescence staining of rAKAV and wtAKAV with mouse monoclonal antibody 2D3 to AKAV N protein

To further prove that AKAV TJ2016 was successfully rescued, the CPE of rAKAV and wtAKAV were observed 48 h post-infection. As shown in Fig. 4, compared with the blank control cells, both rAKAV and wtAKAV infected cells became round, winkled and even shattered. Then, the indirect immunofluorescence assay (IFA) was carried out using the monoclonal antibody against AKAV N protein. As shown in Fig. 4, the similar green fluorescence signals were observed in both rAKAV and wtAKAV infected cells, which means that we have successfully rescued AKAV TJ2016 strain and established the AKAV reverse genetic system.

### Growth kinetics of rAKAV and wtAKAV in BHK-21 cells

**Fig. 5:**
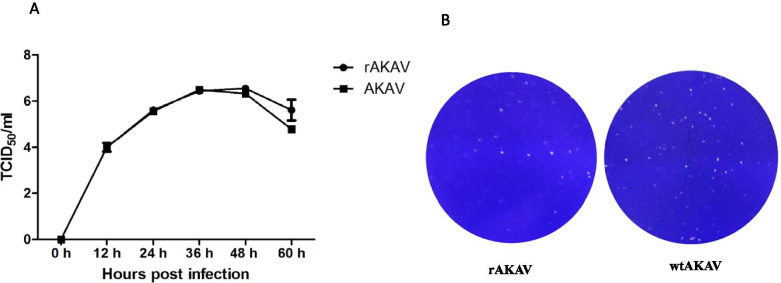
Multi-step growth curves and plaque sizes of rAKAV and wtAKAV on BHK-21 cells

The growth properties of the rAKAV and wtAKAV were investigated by the multi-step growth assay with a multiplicity (MOI) of 0.01. As shown in Fig. 5A, rAKAV exhibited a similar growth kinetic to wtAKAV. The maximum titer of rAKAV and wtAKAV was 3.55 × 10^6^ and 3.2 × 10^6^ TCID_50_/ml, respectively, at 48 h post-infection. Essentially no significant difference in growth kinetics was observed between the wtAKAV and rAKAV. Then, BHK-21 cells were infected with 0.01 MOI of rAKAV and wtAKAV respectively, and the plaque sizes and shapes were confirmed 72 h post-infection. Similar with their growth kinetics, the plaque shape and size of rAKAV also had no significant differences from that of wtAKAV (Fig. 5B).

## Discussion

AKAV is widely prevalent in tropical and temperate climate zones in Africa, Asia, Middle East and Australia [14], and it has caused serious economic losses to animal husbandry and posed a great threat to cattle, sheep and goat breeding. In China, the first detection of the virus could be traced back to 1990s by the Animal Quarantine Institute of Ministry of Agriculture using the neutralization assay. The goat or cattle sera derived from several provinces including Shandong, Hebei, Shaanxi, Gansu, Hunan were detected to be AKAV positive [15]. Since then, many studies proved that in many provinces of China, the neutralizing antibody positive rate of AKAVs in cattle, goats and sheep was higher than 10% [9, 10]. Meanwhile, several AKAV strains have been isolated from various species including mosquitoes, bamboo rat and goat [11–13, 16]. Phylogenetic trees based on the S and M segments sequences revealed that all the above isolates belong to AKAV genogroup I. In our study, phylogenetic analysis based on the S and M segments indicates that the isolated TJ2016 belongs to genogroup II.

AKAV belongs to the Simbu serogroup of Orthobunyaviruses [17]. Because of its segmented viral genome, closely related viruses within the order Bunyavirales can undergo genetic reassortment, with exchange of segments, to produce recombinant genotypes that remain infectious. Phylogenetic studies also suggested that genetic reassortments often occured among viruses of Bunyaviridae [18, 19]. This theoretically mostly occured in vertebrate hosts infected simultaneously with two or more Bunyaviruses, in a manner akin to that documented for influenza viruses [20, 21]. A previous phylogenetic analysis of Schamllenberg virus (SBV), member of the Simbu serogroup, revealed that SBV might be a reassortant virus with the M RNA-segment from Sathuperi virus (SATV) and the S and L RNA-segments from Shamonda virus (SHAV), since SBV sequence shared an 81.8 to 82.2% identity with the SATV M-RNA-segment, 96.4 to 96.7% and 89.5 to 94.1% identities with the SATV S-RNA-segment and L-RNA-segment, respectively [19]. In China, both AKAV genogroup I and genogroup II exist in the clinical goat or cattle farms, which rise the possibility of the reassortant of AKAV.

Reverse genetic systems are useful for studying basic mechanisms as well as practical applications for many kinds of viruses. Previously, the RNA polymerase I was employed to develop the reverse genetic system of OBE-1 strain for AKAV [22]. However, its efficiency for virus rescue was substandard, lack of robust. While, T7 RNA polymerase-based reverse-genetics system has proved to be more effective in rescuing other bunyaviruses [23–25]. In our study, we successfully rescued the AKAV strain TJ2016 using the T7 RNA polymerase-based reverse genetic system. In this system, only three plasmids (TVT7R-S, TVT7R-M, and TVT7R-L) needed and we successfully rescued the TJ2016 strain in all of the three plasmid proportions (1: 1: 1, 0.5: 1: 2 and 0.5: 2: 4) which means that the proportion of TVT7R-S, TVT7R-M, and TVT7R-L plasmids is not severely restricted.

## Conclusions

We successfully isolated a genogroup II AKAV strain TJ2016 and established its T7 RNA polymerase-based rescue genetic system, which could be served as a powerful tool for further AKAV recombination strategies, pathogenesis mechanism and even vaccine studies.

## Methods

### Cells and viruses

Baby hamster Syrian kidney (BHK-21) cells and BSR-T7/5 cells (a BHK-21 derivative cell that stably expresses T7 RNA polymerase) used in this study were stored at the Institute of Animal Inspection and Quarantine, Chinese Academy of Inspection and Quarantine (CAIQ) and maintained on Dulbecco’s modified Eagle’s medium (DMEM) containing 10% fetal bovine serum (FBS) [26]. AKAV strain TJ2016 used in the present study was isolated from the bovine sera stored at our lab which were sent for animal health monitoring before.

### PCR amplification, sequencing and phylogenetic analysis

To define the relationships of the isolated bovine AKAV TJ2016 with other AKAV isolates, phylogenetic trees were constructed based on the S and M ORF of AKAV TJ2016 and the deposited AKAV sequences in the GenBank. Briefly, the viral RNA of TJ2016 was extracted from virus-containing cell culture supernatant using the viral RNA min kit (Qiagen, Hilden, Germany) and stored at − 80 °C. The RT-PCR procedure was performed in a one-tube system for sequencing using the EasyScript All-in-one First-strand cDNA Synthesis SuperMix (TransGen Biotec, Beijing, China) and the primers used for the amplification of AKAV S, M, and L segments were shown in Table 1 (Number 1 to 18). The amplified DNA fragments were purified using an Agarose Gel DNA Extraction Kit (TransGen Biotec) and sequenced by TSINGKE Biological Technology (Nanjing, China). The S and M segment sequences of the Akabane virus strains used for sequence alignment in this study were listed in Table 2. The alignments and the neighbor-joining (NJ) phylogenetic trees were constructed using the MEGA 5.0 software [27]. The branching pattern was statistically evaluated by bootstrap analysis of 1000 replicates.

**Table 2:** Summary of Akabane virus field isolates used in this study

Strain Name	Year	Country	Source	Genogroup	Accession No.	
					S CDS	M CDS
GXLCH02	2016	China	*Rhizomys pruinosus*	Ia	KY381272.1	
NM/BS/1	2016	China	Bovine serum	Ia	KU375444.1	KU375443.1
GXLCH01	2016	China	*Rhizomys pruinosus*	Ia	KY385908.1	KY381277.1
GXLCH04	2016	China	*Rhizomys pruinosus*	Ia	KY381273.1	KY381279.1
GXLCH16-70	2016	China	*Rhizomys pruinosus*	Ia	KY381274.1	KY381280.1
GXLCH70N	2016	China	*Rhizomys pruinosus*	Ia	KY381275.1	KY381281.1
KM-1/Br/06	2006	Japan	Bovine brain	Ib	AB426271.1	AB436954.1
KM-2/Br/06	2006	Japan	Bovine brain	Ib	LC552047.1	LC552048.1
KS-2/Mo/06	2006	Japan	Bovine brain	Ib	AB373232.1	AB373233.1
KSB-3/P/06	2006	Japan	Bovine plasma	Ib	AB426280.1	AB426282.1
AKAV-7/SKR/2010	2010	South Korea	Cattle	Ib	JQ308771.1	JQ308775.1
AKAV-32/SKR/2010	2010	South Korea	Cattle	Ib	JQ308773.1	JQ308777.1
AKAV-17/SKR/2010	2010	South Korea	Cattle	Ib	JQ308772.1	JQ308776.1
Okayama2001	2001	Japan	Bovine plasma	Ib	AB289319.1	AB289322.1
Iriki	1984	Japan	Calf cerebellum	II	AB289321.1	AB297820.1
CY-77	1993	Taiwan	Bovine erythrocyte	II	AB232319.1	AB297851.1
PT-17	1997	Taiwan	Bovine blood	II	AF034940.1	
NT-14	2000	Taiwan	Pig	II	AF529883.1	
FO-90-3	1990	Japan	*Culicoides spp*	II	AB232198.1	AB297831.1
KT3377	1977	Japan	Bovine blood	II	AB000857.1	AB297819.1
AK7	2006	South Korea	Bovine	II	FJ498795.1	FJ498801.1
K0505	2005	South Korea	Bovine	II	FJ498796.1	FJ498800.1
93FMX	1993	South Korea	Bovine	II	FJ498797.1	FJ498799.1
OBE-1	1974	Japan	Aborted fetus brain	II	AB000851.1	AB100604.1
YG-88-2	1988	Japan	Bovine erythrocyte	II	AB232196.1	AB297827.1
ON-89-2	1989	Japan	Bovine erythrocyte	II	AB000864.1	AB29297828.1
NS-88-1	1988	Japan	Bovine erythrocyte	II	AB000864.1	AB297825.1
JaGAr39	1959	Japan	*Aedes vexans*	II	AB000852.1	AB297818.1
JaLAB39	1959	Australia	*Aedes vexans*	II	KR260714.1	KR260715.1
R7949	1968	Australia	*Bos taurus*	III	MH734959.1	MH735016.1
B8935	1968	Australia	*Bos taurus*	III	MH734940.1	AB297848.1
MP496	1972	Kenya	*Anopheles funestus*	IV	AB232320.1	AB297850.1

### Plasmid construction and the generation of rescued virus rAKAV

For the establishment of AKAV reverse genetic system, the full-length sequences of S, M, and L segments were amplified using the primers listed in Table 1 (Number 19 to 24) and the amplified DNA products were cloned into the plasmid TVT7R as described previously [28], which could transcribe the antigenome-sense RNA by T7 RNA polymerase. Briefly, viral RNA was first extracted from virons cultured in the BHK-21 cells using the viral RNA min kit (Qiagen). Then, the viral RNA was reverse-transcribed using the EasyScript All-in-one First-strand cDNA Synthesis SuperMix (TransGen Biotech.) and the full-length sequences of S, M, and L segments were amplified by the KOD Fx Neo polymerase. The plasmid TVT7R and the amplified PCR products were digested by the endonucleases *Bbs*I and *Bsm*BI, respectively, and subsequently ligated using the T4 ligase (TaKaRa Bio.). The concentrations of the successfully constructed S, M, and L recombinant plasmids were measured by the Nanodrop microspectrophotometer (Thermo Fisher Scientific, MA, USA) and then used in a three-plasmid rescue system by transfecting into the T7 RNA polymerase-expressing BSR-T7/5 cells [29]. Briefly, BSR-T7/5 cells were seeded into the six-well plate until the cells covered 80-90% area of the plate, S, M, and L segments (1:1:1) were transfected to the cells by using the Lipofectamine™ 3000 transfection reagent (Thermo Fisher Scientific). Three to 4 days after transfection, the cell supernatants were harvested and added into BHK-21 cells. Then, viruses were plaque purified three times from BHK-21 cells and stored at − 80 °C until used [30].

### Indirect immunofluorescence assay

For the immunofluorescence imaging of AKAV virons, BHK-21 cells were grown in six-well plates to 80-90% confluence, then were infected with wtAKAV or rAKAV for 48 h at 37 °C. The supernatants were removed and cells were fixed with 3.7% paraformaldehyde for 10 min and permeabilized with 0.1% Triton X-100 in phosphate buffer saline (PBS) containing 2% bovine serum albumin (BSA) for 10 min followed by blocking with 2% BSA in PBS for 30 min. Then the cells were incubated with AKAV anti-N monoclonal antibody (diluted by 1:1000, produced by our lab) as the primary antibody at 37 °C for 1 h and then washed with PBS for three times. Stained with FITC-conjugated goat anti-mouse antibody (diluted by 1: 500, TransGen Biotech.) as the secondary antibody at 37 °C for 1 h, the cell-cultured plates were washed with PBS for 3 times and were observed under the Invitrogen EVOS FL cell fluorescence imaging system (Thermo Fisher Scientific).

### Viral plaque assay

BHK-21 cells in six-well plates were infected with rAKAV or wtAKAV at MOI of 0.01. for 1 h at 37 °C. The medium containing the unbound viruses were removed, and the plates were washed three times with PBS before being overlaid with DMEM containing 0.5% methylcellulose. At 72 h post-infection, the medium was removed and the plaques were stained with 3.7% paraformaldehyde containing 0.1% crystal violet.

### Growth kinetics of wtAKAV and rAKAV

To compare the growth kinetics of wtAKAV and rAKAV, BHK-21 cell monolayer was infected with AKAVs at a MOI of 0.01. After 1 h at 37 °C incubation, the unbound viruses were removed and washed with PBS for three times, and serum-free DMEM was added. At different times post-infection, cells were harvested and titrated using tissue culture infectious dose 50 (TCID_50_) with BHK-21 cells [31].

### Statistical analysis

All data was processed with GraphPad Prism 6 (GraphPad Software Inc.). A student *t-*test was used to analyze the difference between the values of two groups. A value of *p* < 0.05 was considered statistically significant.

## Supplementary Information

**Additional file 1: Figure S1.** Sequence aligment of rAKAV and wtAKAV. **Figure S2.** The sequence of rAKAV S segment. **Figure S3.** The plague of rAKAV and wtAKAV. **Figure S4.** The PCR result of rAKAV and wtAKAV.

## Data Availability

The datasets used and/or analyzed during the current study are available from the corresponding author on reasonable request.

## Abbreviations

AH: Arthrogryposis hydranencephaly
AKA: Akabane disease
AKAV: Akabane disease virus
ORF: Open reading frame
CPE: Cytopathic effect
MOI: Multiplicity
TCID_50_: Tissue culture infectious dose 50
BSA: Bovine serum albumin
SBV: Schmallenberg virus
FBS: Fetal bovine serum
DMEM: Dulbecco’s modified eagle medium
PBS: Phosphate-buffered saline
IFA: Indirect immunofluorescence assay
BHK: Baby hamster syrian kidney
RT-PCR: Reverse transcription polymerase chain reaction
