# Supplementary material for: Characterization and reverse genetic establishment of cattle derived Akabane virus in China
Source: BMC Vet Res. 2021 Nov 15;17:349. doi: 10.1186/s12917-021-03054-x (PMC8591888; doi:10.1186/s12917-021-03054-x)

Figure S1 Sequence aligment of rAKAV and wtAKAV


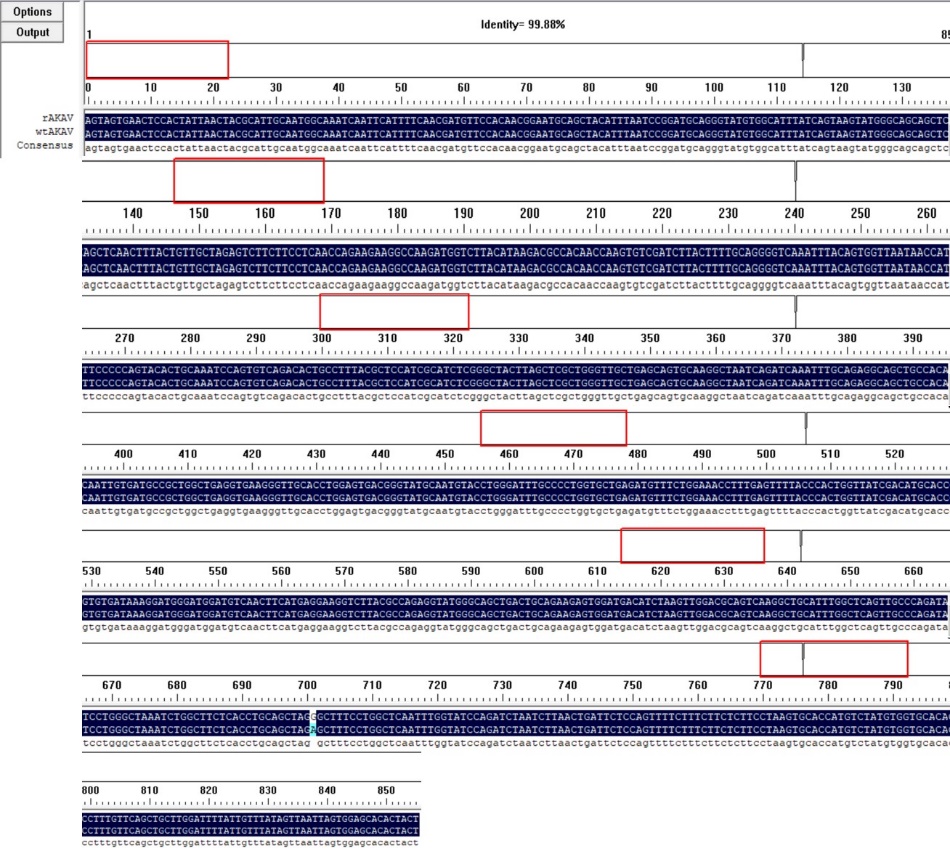


Figure S2 The sequence of rAKAV S segment


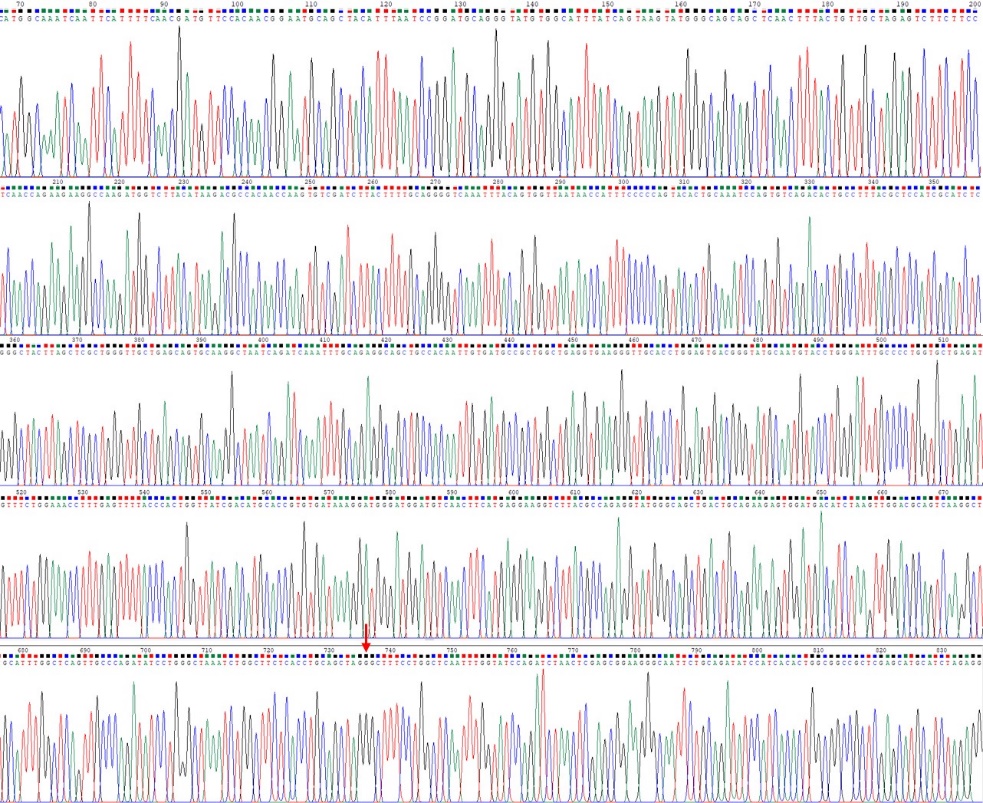


Figure S3 The plague of rAKAV and wtAKAV


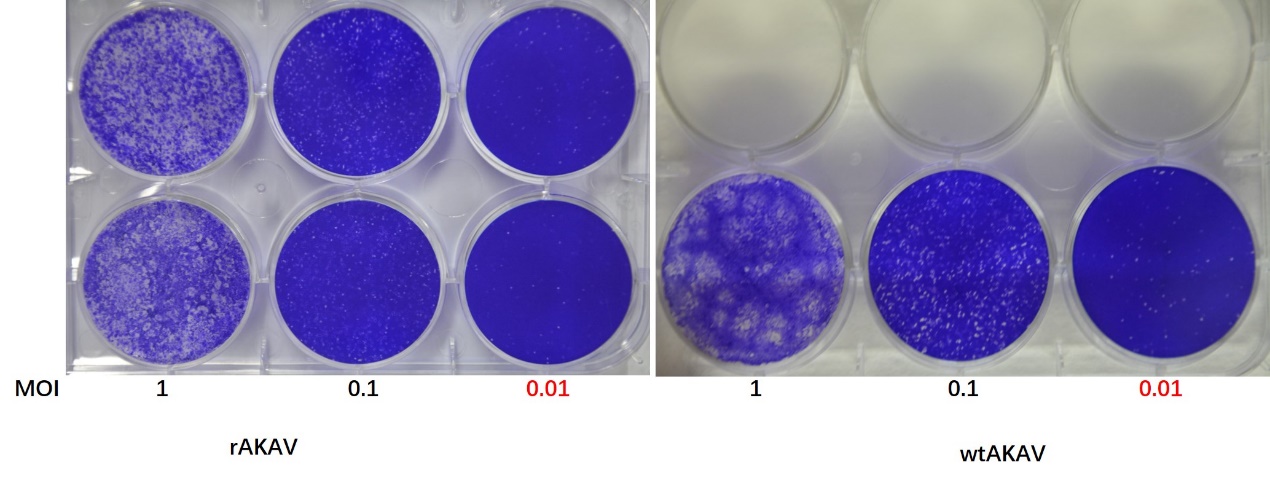


Figure S4 The PCR result of rAKAV and wtAKAV


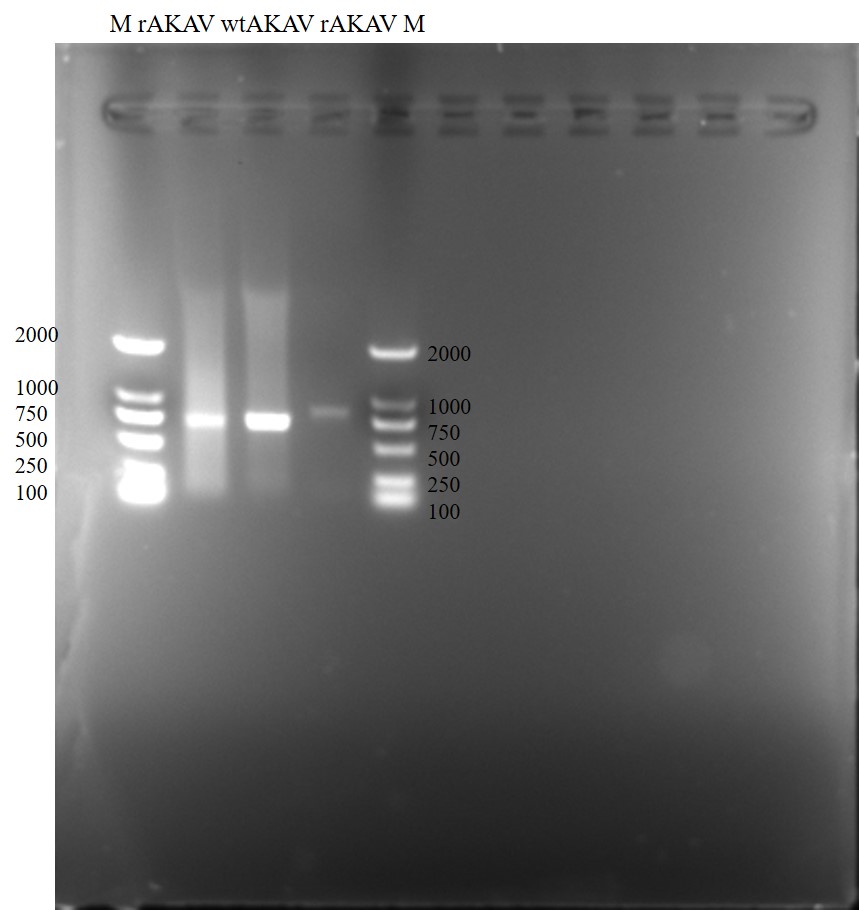

Supplement: Supplementary file 1 — Additional file 1: Figure S1. Sequence aligment of rAKAV and wtAKAV. Figure S2. The sequence of rAKAV S segment. Figure S3. The plague of rAKAV and wtAKAV. Figure S4. The PCR result of rAKAV and wtAKAV. [file 12917_2021_3054_MOESM1_ESM.docx]
